# Dynamic Response of Polyindole Coated Zinc Ferrite Particle Suspension under an Electric Field

**DOI:** 10.3390/ma15010101

**Published:** 2021-12-23

**Authors:** Su Hyung Kang, Hyoung Jin Choi

**Affiliations:** 1 Department of Polymer Science and Engineering, Inha University, Incheon 22212, Korea; 22201158@inha.edu; 2 Program of Environmental and Polymer Engineering, Inha University, Incheon 22212, Korea

**Keywords:** electrorheological, core-shell, zinc ferrite, polyindole

## Abstract

ZnFe_2_O_4_ particles initially synthesized through a simple solvothermal method were coated using polyindole (PIn) to prepare an actively controllable core-shell typed hybrid material under both electric and magnetic fields. An advantage of this process is not needing to add the stabilizers or surfactants commonly used for uniform coating when synthesizing core or shell-structured particles. The synthesized ZnFe_2_O_4_/PIn particles have a lower density than conventional magnetic particles and have suitable properties as electrorheological (ER) particles. The expected spherical shape of the particles was proven using both scanning electron microscopy and transmission electron microscopy. The chemical characterization was performed using Fourier-transform infrared spectroscopy and X-ray diffraction analysis. To analyze the rheological properties, a ZnFe_2_O_4_/PIn based suspension was prepared, and dynamic rheological measurements were performed for different electric field strengths using a rotary rheometer. Both dynamic and elastic yield stresses of the ER fluid had a slope of 1.5, corresponding to the conductivity model. Excellent ER effect was confirmed through rheological analysis, and the prepared ER fluid had a reversible and immediate response to repeated electric fields.

## 1. Introduction

Smart materials whose properties can be tuned by various external stimuli (physical, chemical, or biological) have received widespread interest in many engineering fields [1]. Among these, electrorheological (ER) and magnetorheological (MR) fluids are actively controllable systems that respond to external electric and magnetic fields, respectively. ER fluids are systems in which electrically polarizable particles are homogeneously suspended in an in-active carrier medium. Without an external electric field strength (*E*), the suspensions are in a liquid-like form, but when the *E* is applied, the suspension becomes solid [2]. This state change occurs when a fine filament structure is formed as a result of the dipole-dipole interactions of the polarized particles in the electric field [3]. The degree of structural change and rapid rheological behavior switching in ER fluids can be tuned by the external *E*. Consequently, actively controllable ER fluids are attracting attention for use in applications in various technical fields such as polishing devices, dampers, and brakes [4]. Among the various conductive polymers that have been used as polarizable particles, polyindole (PIn) [5,6,7,8] has the advantage of superior redox activity and thermal stability compared to typical conducting polymers such as polyaniline (PANI) and polypyrrole. In contrast to the ER fluid, MR fluid is a suspension in which soft-magnetic particles are randomly dispersed in a matrix liquid; this enables the fluid physical structure to be changed in a reversible nature as described above, with the presence of an external magnetic field [9,10]. By coating a conductive polymer on magnetic particles used as MR materials, electro-magnetorheological (EMR) particles, which are dual-responsive materials that respond in both magnetic and electric fields, can be synthesized. Furthermore, this ‘core-shell’ structure can overcome the precipitation problem because the low-density conductive polymer plays a role in lowering the density of magnetic particles [11]. Micron-sized carbonyl iron (CI) particles are magnetic and have a high M_S_ value and soft magnetic properties favorable for MR, which can be redistributed into a medium when the magnetic field is removed [12,13]. However, the CI has a high density of approximately 7.9 g/cm^3^ and can cause severe sedimentation stability problems due to the high-density difference within the medium of MR fluids. In addition, because its oxidation stability is very unstable, various methods have been proposed to overcome the disadvantages of using coating agents or additives [14,15]. Spinel ferrite with a lower density of approximately 4.5 g/cm^3^ could be a better alternative for Cl particles [16]. Among spinel ferrites, zinc ferrite (ZnFe_2_O_4_) is composed of a cubic spinel structure in which Zn^2+^ ions seize a tetrahedral position and Fe^3+^ ions fill an octahedral position. ZnFe_2_O_4_ can be selected as a magnetic particle that serves as a stable core for fluid because it has superparamagnetic properties with sufficiently high M_S_ and low remanent magnetization [17,18]. Among the various synthetic methods [19] for the production of ZnFe_2_O_4_, the solvothermal process does not require precursor preparation and additional annealing and proceeds in one step. This method allows precise fabrication and is nontoxic and economical [20].

In this study, a conductive PIn polymer was coated on the surface of magnetic ZnFe_2_O_4_ particles for the synthesis of a dual-responsive EMR fluid. Composite particles with ZnFe_2_O_4_ as the core and PIn as the shell were fabricated via a sequential solvo-thermal and an oxidation polymerization process, respectively. Acid treatment was performed on the synthesized ZnFe_2_O_4_ surface using an HCl solution. PIn was uniformly coated through hydrogen bonding interactions with the H+ groups that form on the ZnFe_2_O_4_ surface. No additional surfactants or stabilizers were used during the PIn coating process. By combining magnetic particles and conductive polymers, the overall density of the particles became lowered. A smart fluid with improved sedimentation stability and reactivity in magnetic and electric fields was prepared. This study investigates the ER effect through various dynamic tests in the presence of an electric field. While its fabrication and dual performance of the EMR fluid has been reported recently [21], in this work, we put our emphasis on its more detailed dynamic ER response under an applied *E* along with its dielectric behavior.

## 2. Experimental

### 2.1. Materials and Synthesis

To synthesize zinc ferrite particles, iron chloride hexahydrate (FeCl_3_.6H_2_O) (Sigma-Aldrich, St. Louis, MO, USA) and ZnCl_2_ (Yakuri Chem., Kyoto, Japan) served as providers of Fe and Zn ions, respectively. Ethylene glycol (Daejung Chem., Siheung, Gyeonggi-do, Korea), which is important in ferrite formation as a reducing agent, was also used [22]. Furthermore, polyethylene glycol (PEG 400, MW = 400 g/mol, Duksan Chem.) as a surfactant and C_2_H_3_NaO_2_ (Sigma-Aldrich, USA) as an electrostatic stabilizer were used to prevent particle agglomeration in the liquid-phase process [23]. The materials for magnetic particle coating were indole (Sigma-Aldrich) as a shell monomer, ammonium persulfate (≥98% purity, Daejung Chem., Siheung, Gyeonggi-do, Korea) as an initiator, and HCl (0.1 mol/L or 1 mol/L, 35%, DukSan Chem., Ansan, Gyeonggi-do, Korea) as a dopant. Ethyl alcohol (Samchun Pure Chem., Seoul, Korea) and deionized water were used as the solvent and cleansing solvent, respectively.

#### 2.1.1. Synthesis of ZnFe_2_O_4_

Spherical ZnFe_2_O_4_ particles were produced via a solvothermal process. We performed magnetic stirring to dissolve 6.75 g of FeCl_3_.6H_2_O and 1.7 g and zinc chloride in 250 mL of ethylene glycol solvent. The molar ratio of FeCl_3_.6H_2_O to zinc chloride was 2:1. After sufficient magnetic stirring, 18 g of sodium acetate and 5 g of PEG 400 were slowly poured into the solution and stirred vigorously. The evenly mixed solution was placed in an autoclave (Teflon-lined stainless steel) and heated for 12 h in an oven set at 200 °C. The mixture was then cooled at room temperature, and the product was gathered using a magnet and cleared several times with ethyl alcohol and deionized water. The brown particles were dried in a vacuum oven at 60 °C for one day.

#### 2.1.2. Fabrication of ZnFe_2_O_4_/PIn

The ZnFe_2_O_4_ particles were used as the core material, and PIn was coated onto these core ZnFe_2_O_4_ particles as a shell through oxidation polymerization. We added 1 g of ZnFe_2_O_4_ particles to 400 mL of HCl (0.1 M) solution and dispersed the particles in a sonication bath for 30 min. The solution was mixed at 5 °C for 12 h, and then the solvent was removed using a magnetic bar to obtain acidified ZnFe_2_O_4_ particles. Next, 1 g of indole and 100 mL of ethyl alcohol were added to the reactor, and the mixture was agitated slowly at 5 °C for 10 h. Subsequently, 2.282 g solution of ammonium persulfate (APS) dissolved in 20 mL of HCl (1M) solution was slowly poured into the mixture. After mechanical agitation at 5 °C for 12 h, the final products were collected using an external magnet and cleaned with ethyl alcohol and deionized water. The green product was obtained after drying in a vacuum oven.

**Scheme 1:**
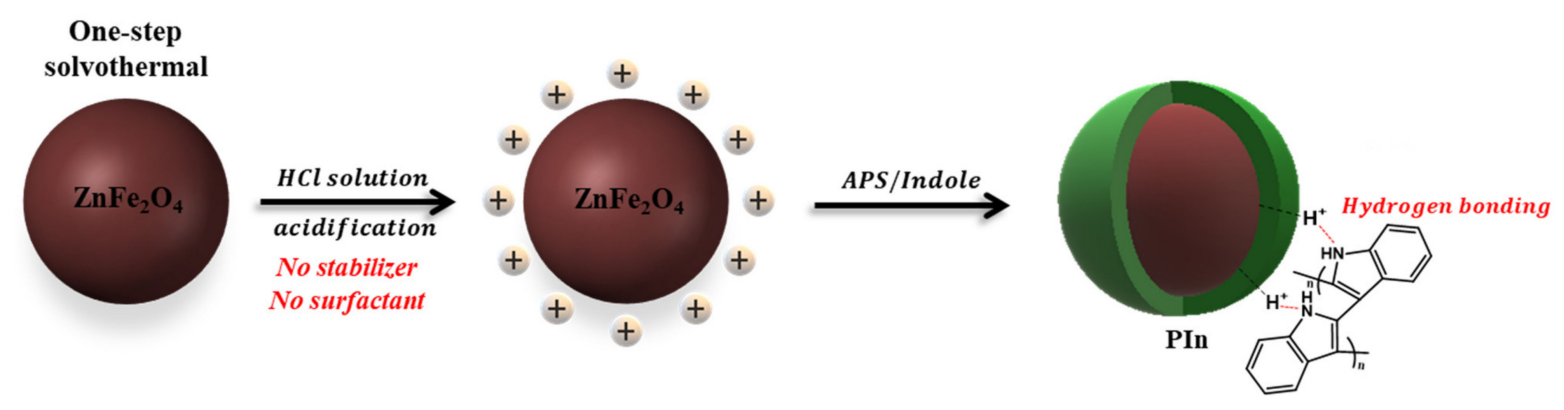
Schematic diagram of the synthesis mechanism of ZnFe_2_O_4_/PIn particles.

The synthesis mechanism of ZnFe_2_O_4_/PIn is shown in Scheme 1. ZnFe_2_O_4_/PIn composites with a core-shell structure were manufactured through a synthesis method without surfactants and stabilizers. By maintaining the ZnFe_2_O_4_ particles synthesized by the solvothermal method in the HCl solution, the surface of the magnetic particles was acidified and protonated with H+ ions. Then, oxidative polymerization was performed using an indole monomer and an initiator in the next step. Here, indole monomer containing nitrogen element tends to be adsorbed on the surface of magnetic particles through hydrogen bonding with H+ ions on the surface of ZnFe_2_O_4_. In addition, indole monomer present around the ZnFe_2_O_4_ particles forms PIn shell by nucleating and growing on the surface of magnetic particles through an oxidative polymerization process. Through this mechanism, ZnFe_2_O_4_/PIn particles with a core-shell structure are formed [24,25].

#### 2.1.3. Fabrication of ER Fluid

For ER characterization, a smart fluid responding to electric fields was prepared by suspending the fabricated ZnFe_2_O_4_/PIn particles in silicone oil (100 cSt, 5 vol%) under sufficient sonication. As a pretreatment, ZnFe_2_O_4_/PIn was de-doped using sodium hydroxide (NaOH) (1 M). The electrical conductivity of the de-doped products was 5.13 × 10^−8^ S/cm, which is an appropriate value for ER fluid preparation.

### 2.2. Characterization

The shape and size of the ZnFe_2_O_4_/PIn microspheres were analyzed using scanning electron microscopy (SEM) (SU-8010, Hitachi, Japan) at 15 kV and a particle size analyzer (ELS-8000, Otsuka, Japan) by measuring the scattering intensity of the laser by the particles in the sample, respectively. Additionally, the morphology of ZnFe_2_O_4_/PIn was examined using a transmission electron microscopy (TEM) (CM-220, Phillips, Amsterdam, The Netherlands). The chemical structure and composition of the particles were verified by Fourier transform infrared spectroscopy (FT-IR) (VER-TEX 80 V, Bruker, Berlin, Germany). X-ray diffractometry (XRD) (DMAX-2500, HORIBA, Kyoto, Japan) was performed to analyze the structure and phase composition of the particles. The density of ZnFe_2_O_4_/PIn measured using a gas pycnometer (AccuPyc 1330, Micromeritics, Norcross, CA, USA) was 3.01 g/cm^3^. In addition, the electrical conductivity was determined using a resistivity meter (Loresta-GP MCP-T610, Mitsubishi Chem., Tokyo, Japan). The dynamic oscillation test of the ER fluids was performed using a Couette-cell rheometer (MCR 302, Anton-Paar, Graz, Austria). In the rheological test, a concentric cylinder (CC17) geometry was used to measure the ER properties. The dielectric properties in the frequency range of 20 to 10^6^ Hz were analyzed using an LCR meter (Agilent HP 4284A, HP, USA).

## 3. Results and Discussion

**Figure 1:**
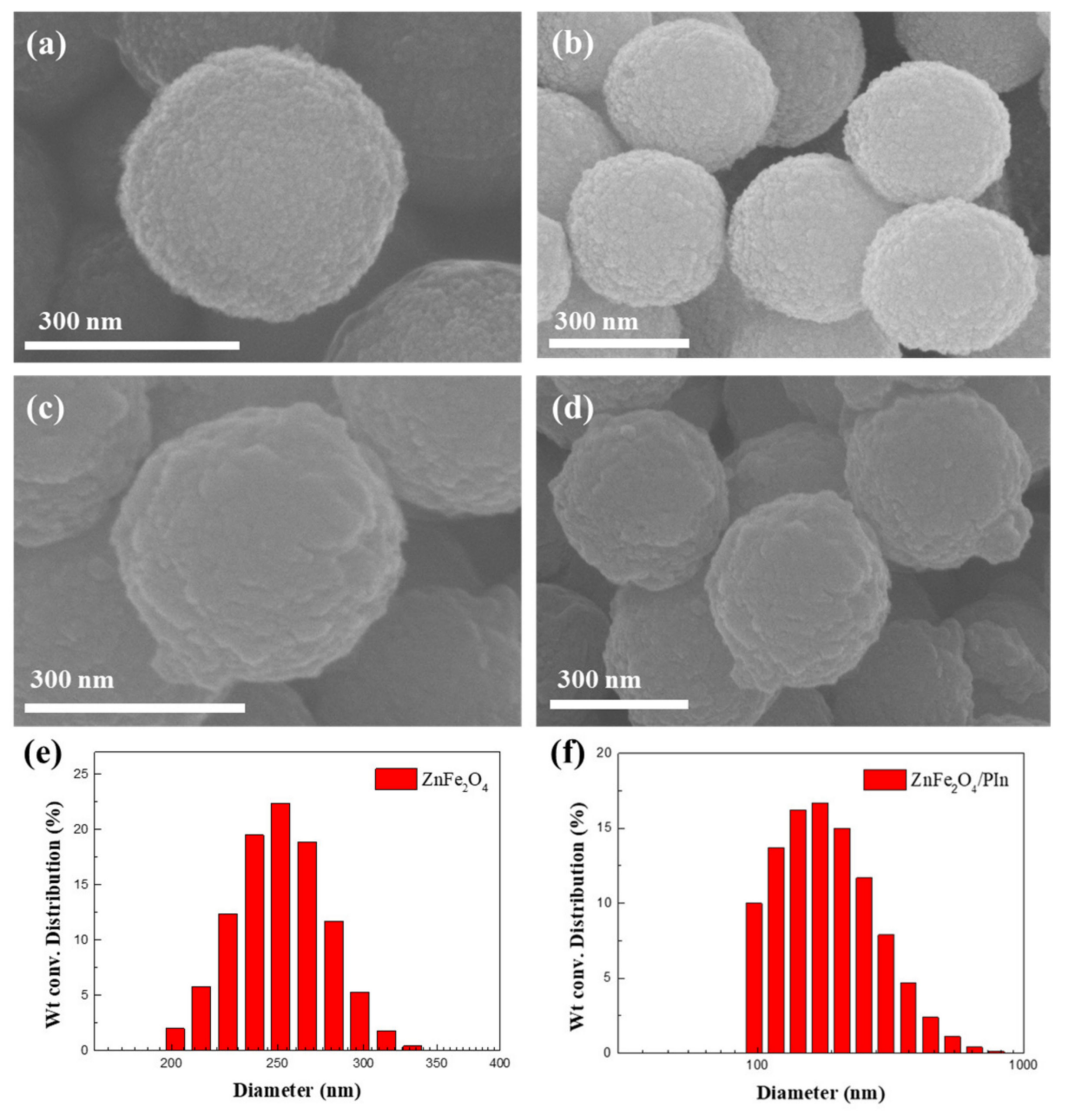
HR-SEM and size distribution images of the ZnFe_2_O_4_ (**a**,**b**,**e**) and ZnFe_2_O_4_/PIn core/shell particles (**c**,**d**,**f**).

Figure 1 presents SEM images of (a, b) ZnFe_2_O_4_ and (c, d) ZnFe_2_O_4_/PIn particles and shows that the ZnFe_2_O_4_ synthesized through the solvothermal method has a spherical shape. As the core material preceded the PIn coating, surface changes were observed on the rough ZnFe_2_O_4_ particle surface. In addition, the PIn coated ZnFe_2_O_4_ particles maintain a spherical shape similar to the particles before polymerization, thereby confirming a uniform coating. Figure 1e,f shows the particle size distribution of ZnFe_2_O_4_ and ZnFe_2_O_4_/PIn, respectively. The average diameter of the ZnFe_2_O_4_ and PIn-coated particles is approximately 256.1 and 274.6 nm, respectively. In addition, it can be also noted that some fractions with a size below 100 nm in Figure 1f might be the uncoated pristine PIn particles even though the synthesized hybrid particles were separated using a magnet. 

**Figure 2:**
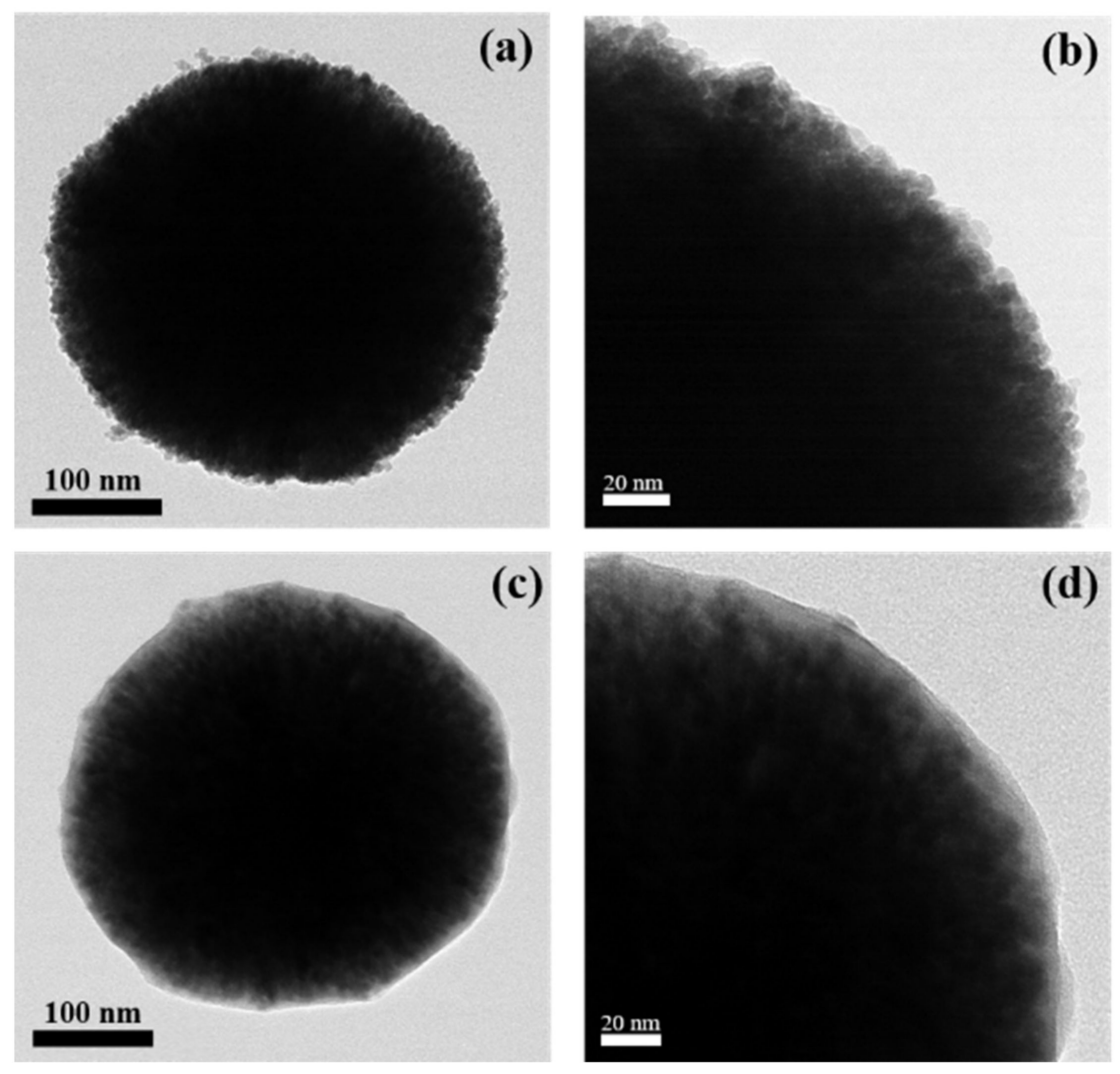
TEM images of the ZnFe_2_O_4_ (**a**,**b**) and ZnFe_2_O_4_/PIn core/shell particles (**c**,**d**).

Figure 2 presents TEM images of (a, b) ZnFe_2_O_4_ and (c, d) ZnFe_2_O_4_/PIn core/shell particles. Figure 2a,b depicts the spherical morphology and rough surface of the core material ZnFe_2_O_4_ particles. Figure 2c,d depicts a uniform cover-like PIn coating on the surface of the ZnFe_2_O_4_. The shell thickness is approximately 15 nm, which is similar to the average particle diameter data measured by the particle size analyzer in Figure 1e,f.

**Figure 3:**
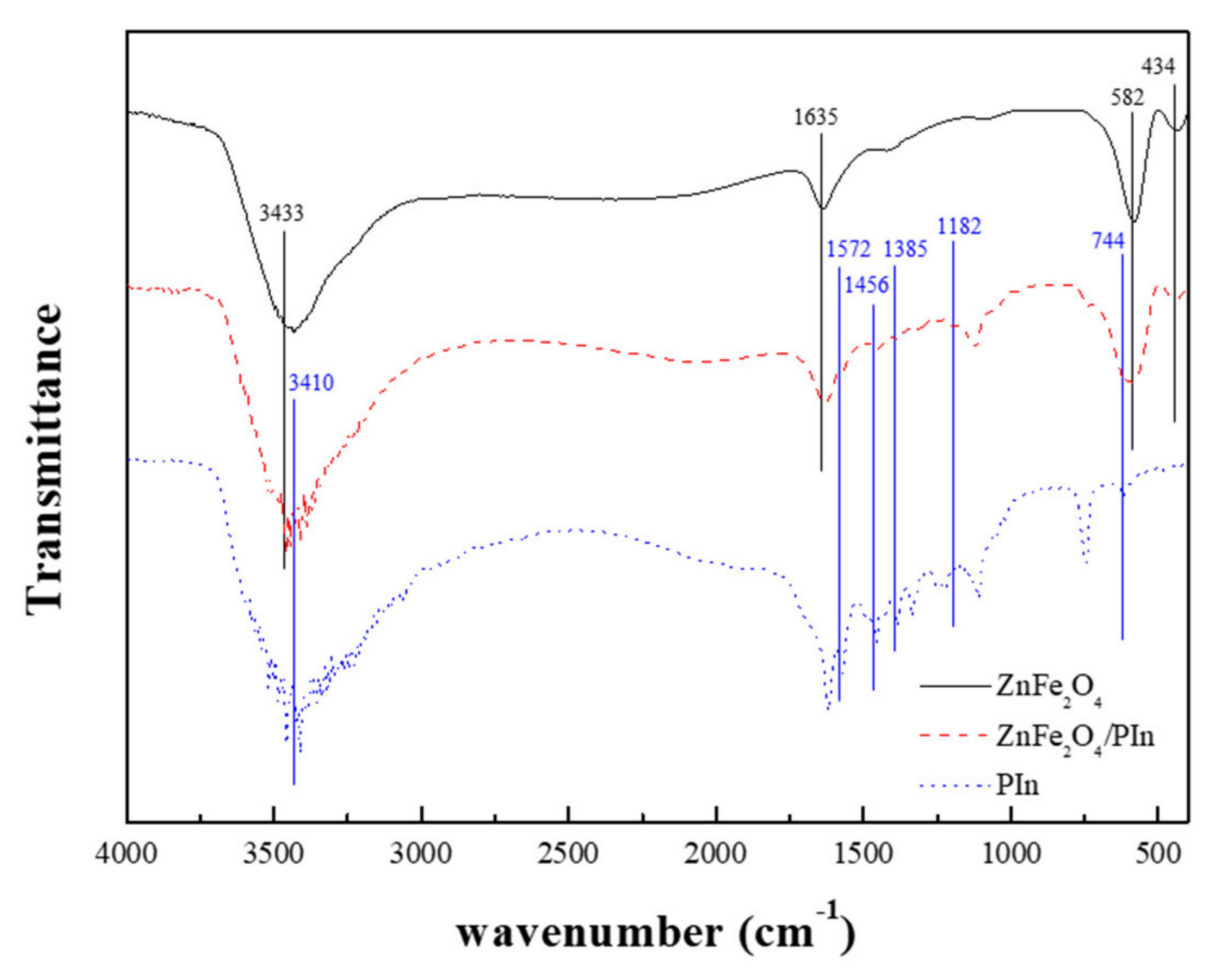
FT–IR spectra of the pure ZnFe_2_O_4_, ZnFe_2_O_4_/PIn and pure PIn particles.

Figure 3 presents the FT-IR spectra of each of ZnFe_2_O_4_, ZnFe_2_O_4_/PIn, and PIn particles with a wave number from 400 to 4000 cm^−1^. In the spectra of the core ZnFe_2_O_4_, signals at 3433 and 1635 cm^−1^ were present due to the stretching vibration of the OH group. Signals at 582 and 434 cm^−1^ show the stretching vibrations of Fe-O and Zn-O, respectively [26]. In the spectra of PIn, a peak at 3410 cm^−1^ indicates N-H stretching bonds, and peaks at 1618 and 1572 cm^−1^ indicate aromatic C=C stretching bonds. Peaks at 1456 and 1385 cm^−1^ indicate the stretching bonds of C-N and C=N, respectively. In addition, in and out-plane C-H bonds of the benzene ring were detected at 1182 cm^−1^ and 744 cm^−1^, respectively [27]. The peaks identified in ZnFe_2_O_4_ and PIn were also detected in ZnFe_2_O_4_/PIn core/shell particles, confirming that ZnFe_2_O_4_/PIn particles were well synthesized.

**Figure 4:**
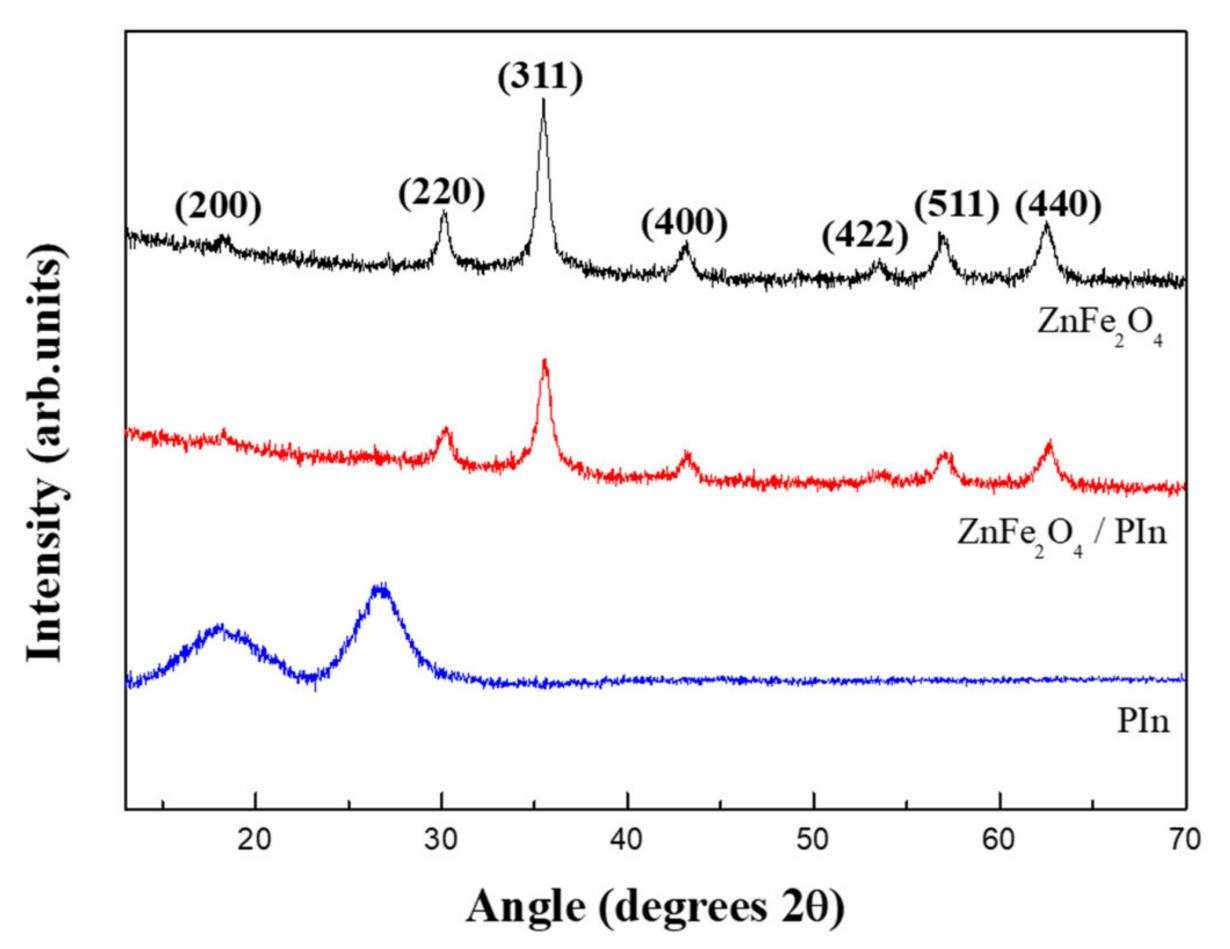
XRD patterns of the ZnFe_2_O_4_, ZnFe_2_O_4_/PIn and pure PIn particles.

Figure 4 shows the XRD pattern for the analysis of the crystal structures of ZnFe_2_O_4_, ZnFe_2_O_4_/PIn, and PIn particles. The diffraction peaks of spinel ZnFe_2_O_4_ particles were detected at 2θ = 18.2°, 30.1°, 35.4°, 43.1°, 53.5°, 56.9°, and 62.5°, which correspond to the (200), (220), (311), (400), (422), (511), and (440) crystal planes, respectively [16]. In contrast, the XRD pattern for PIn exhibits two major peaks. One broad peak appears at 18.0° owing to the amorphous structure, and the second peak appears at 26.4° owing to the partial crystallinity of PIn [28]. As shown in Figure 4, the diffraction peaks of ZnFe_2_O_4_/PIn are almost the same as those of ZnFe_2_O_4_, demonstrating that the principal influence on ZnFe_2_O_4_/PIn crystallinity is the ZnFe_2_O_4_. The reason why no significant signal originated from PIn compound in the ZnFe_2_O_4_/PIn was observed from the XRD might be due to the fact that the coating thickness of PIn was about 15 nm, which is very thin compared to the size of the ZnFe_2_O_4_ core. In addition, it could also be explained because the crystallinity of ZnFe_2_O_4_ metal oxide particles is very high compared to that of the polymer [29].

**Figure 5:**
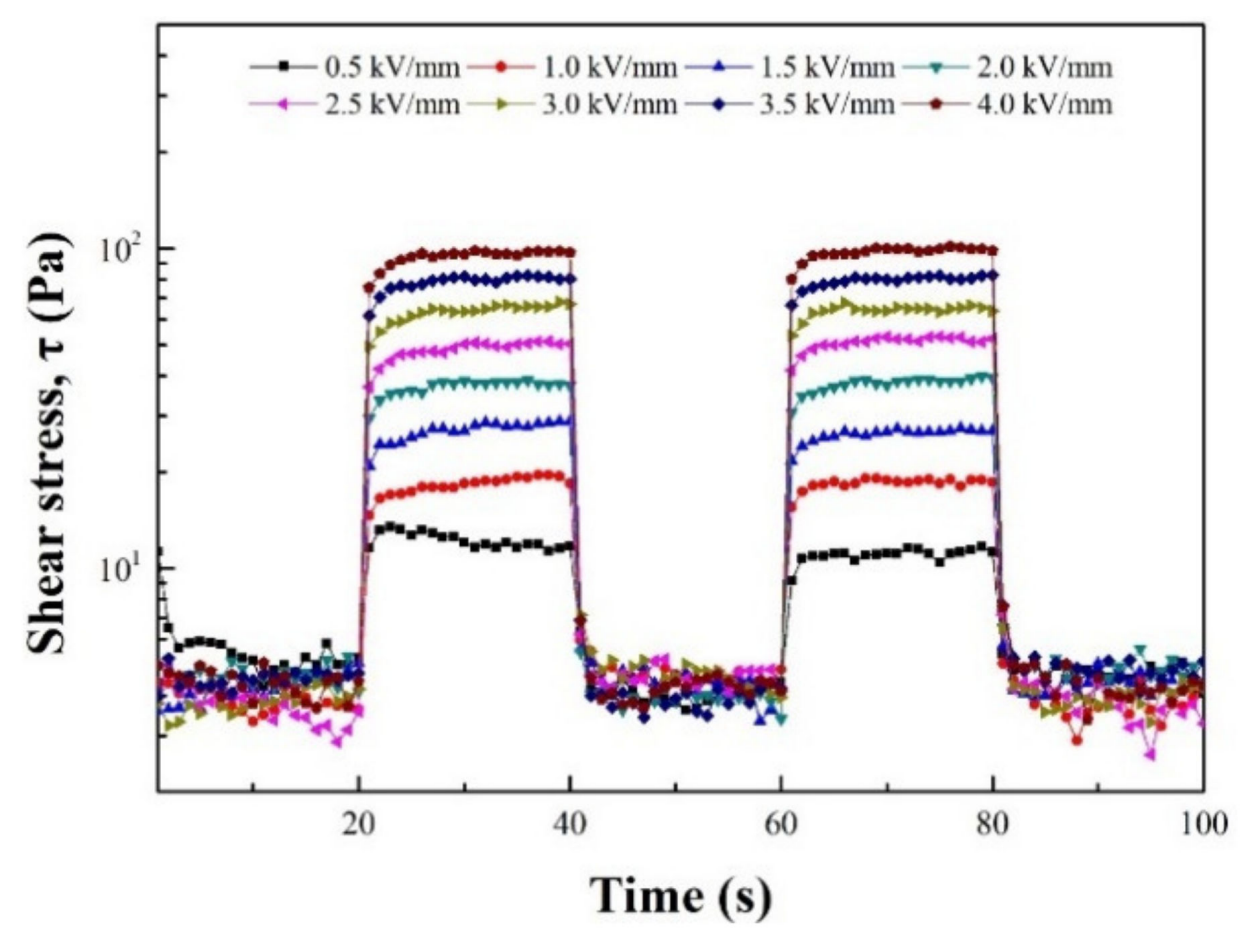
Shear stress reversibility as a function of time for periodic deformation over 20 s intervals of ZnFe_2_O_4_/PIn particle-based ER fluids under various electric fields.

The CC17 geometry used for ER measurement was 16.66 mm in diameter for the bob and 18.08 mm in diameter for cup. Figure 5 demonstrates the reversible response of the shear stress of ZnFe_2_O_4_/PIn particle-based ER fluid when alternately turning the electric field on and off at different strengths. The shear stress increased immediately when the *E* was applied to the fluid and maintained a steady-state value whilst the electric field remained constant and present. Conversely, without the *E*, the shear stress returned to its initial low value and the hysteresis behavior disappeared.

**Figure 6:**
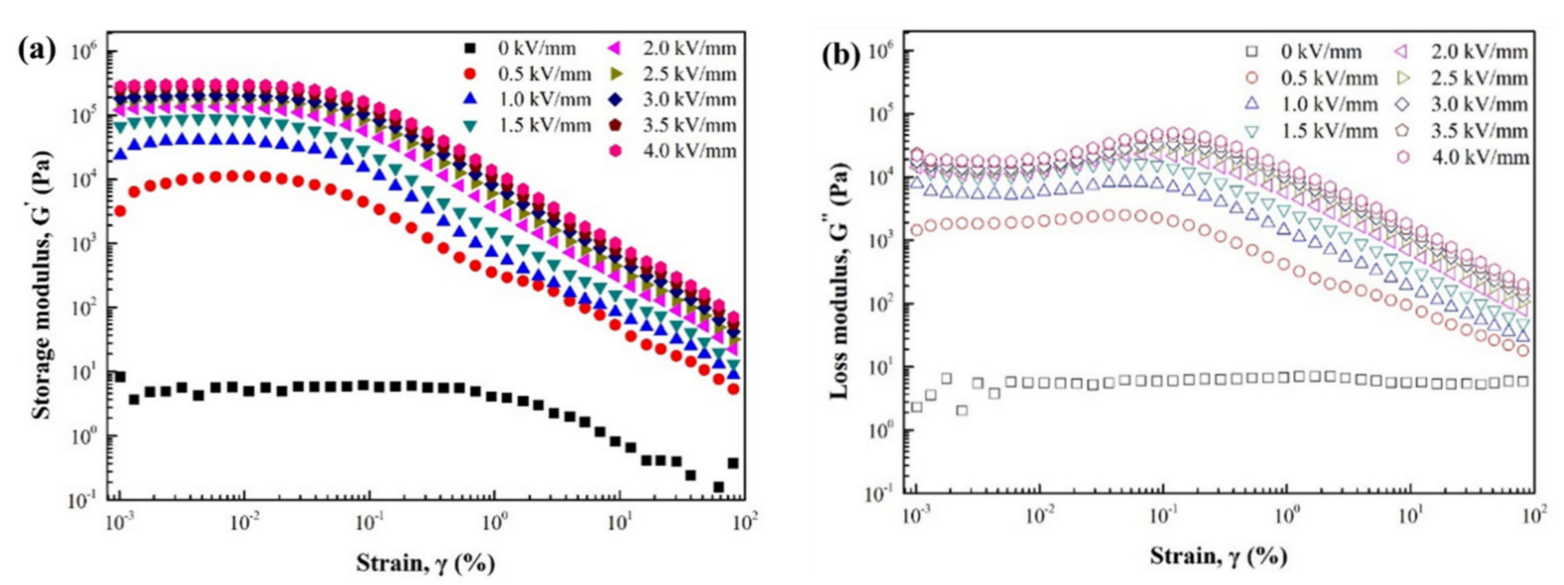
Storage modulus (**a**) and loss modulus (**b**) as a function of strain (%) through dynamic amplitude sweep test of ZnFe_2_O_4_/Pin-based ER fluid under electric field strength.

Figure 6 shows that the (a) storage (G′) and (b) loss modulus (G″) behavior changes with a shear strain (γ). At an angular frequency (ω) of 6.28 rad/s, the electric field gradually increased from 0 to 4 kV/mm in 0.5 kV/mm steps. Without the *E*, the ER sample behaves like a liquid where the G″ is greater than the G′, and both values are lower than those under the electric field. When the *E* is applied, the G′ is more dominant than the G″ at small strain amplitudes at any field strength. In addition, the modulus remained constant, which was defined as the region of the linear viscoelasticity (LVE) region. As the strain increased, the modulus decreased rapidly. As the G′ began to exceed G″, the solid-like behavior was changed to liquid-like behavior. This is the result of the chain structure induced by the applied *E* exceeding the strain it can withstand [30].

**Figure 7:**
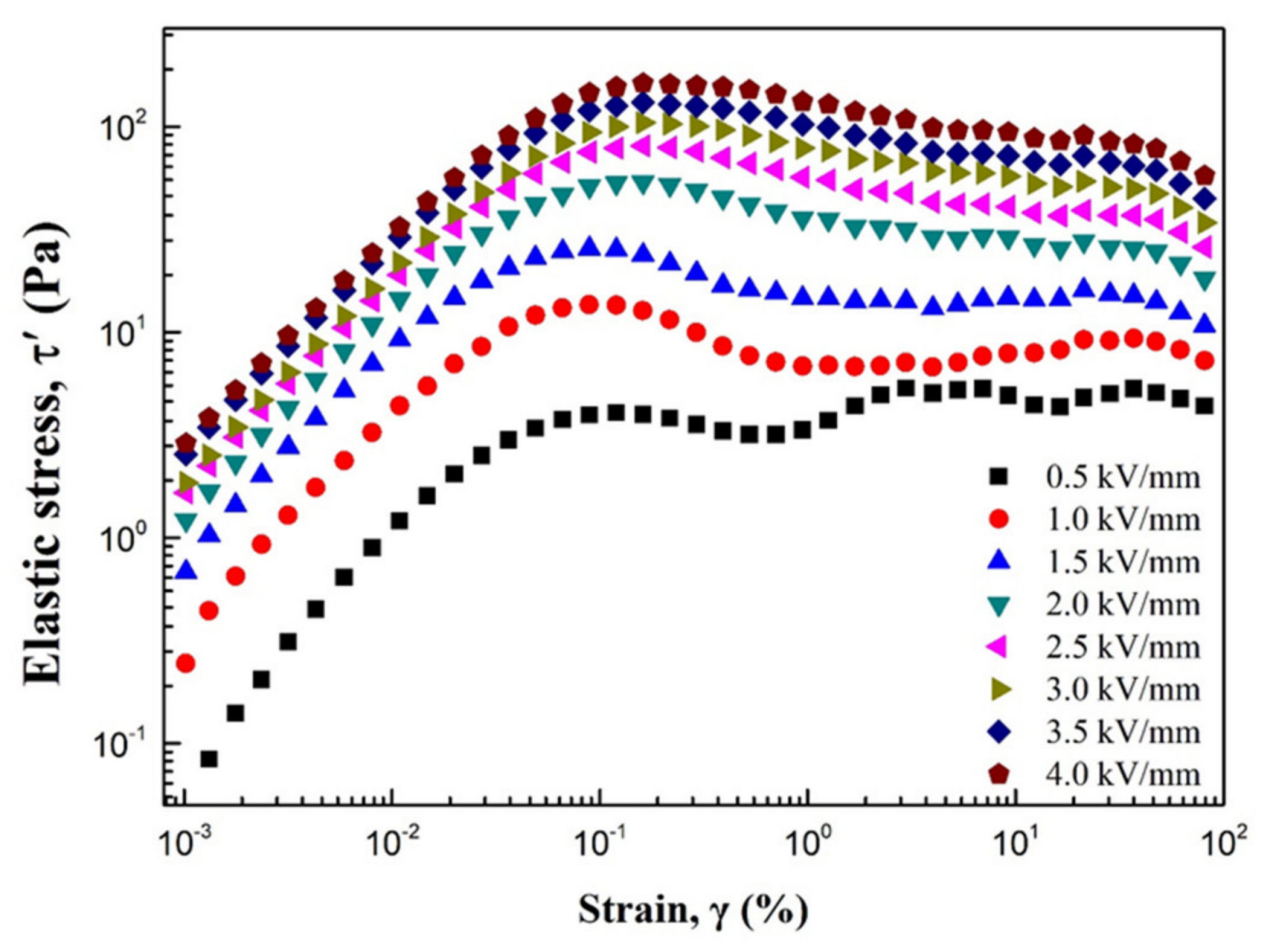
Elastic stress as a function of strain (%) of ZnFe_2_O_4_/PIn particles-based ER fluid with varying electric field strengths.

Figure 7 demonstrates the elastic stress (τ′) under an electric field according to the shear strain. τ′  is calculated using Equation (1) using the storage modulus measured in a dynamic amplitude test [31].

(1) τ′=G′γ

Elastic stress can explain the elastic collapse of the chain structure in which the stimulus-responsive particles are aligned by an external field. As shown in Figure 7, $\tau^{\prime }$ increases linearly in the range of small strain amplitudes. At such amplitudes, even if shear force is applied to the chain structure formed between ZnFe_2_O_4_/PIn particles, elastic structure recovery is possible, maintaining reversible deformation. In contrast, the slope of $\tau^{\prime }\;$ gradually decreases at high strain amplitudes, confirming the presence of maximum elastic stress. The maximum elastic stress is defined as the elastic yield stress and represents the maximum resistance that the internal chain structure of the ER fluid can withstand. If a stress exceeding the maximum elastic stress is applied to the ER fluid, the chain structure cannot be restored elastically [32].

**Figure 8:**
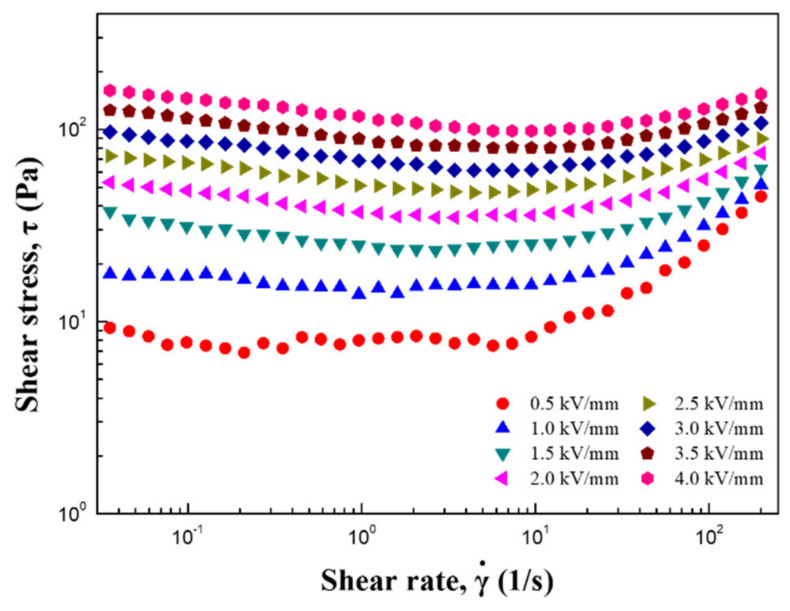
Shear stress curves of ZnFe_2_O_4_/PIn-based ER fluid as a function of shear rate under electric field strengths (Extracted from Ref. [21], © 2021, IEEE).

Flow test of ZnFe_2_O_4_/PIn particle-based ER fluid was also performed using a controlled shear rate test method. Figure 8 represents the shear stress as a function of shear rate applied where the external electric field was gradually increased from 0.5 to 4.0 kV/mm as extracted from our previous report [21]. As the electric field was applied, each shear stress exhibits a relatively constant value over a certain shear rate range along with a yield stress. This yield stress at a zero-shear rate limit is defined as a dynamic yield stress (τ_dy_). This value implies the minimum value of shear stress required for ZnFe_2_O_4_/PIn particles-based ER fluid to start flow. As the electric field strength increases, the shear stress value at the zero-shear rate increases more because the harder chain structure is formed by the influence of electrostatic interaction between interparticles, which is stronger than the hydrodynamic force of the ER fluid.

**Figure 9:**
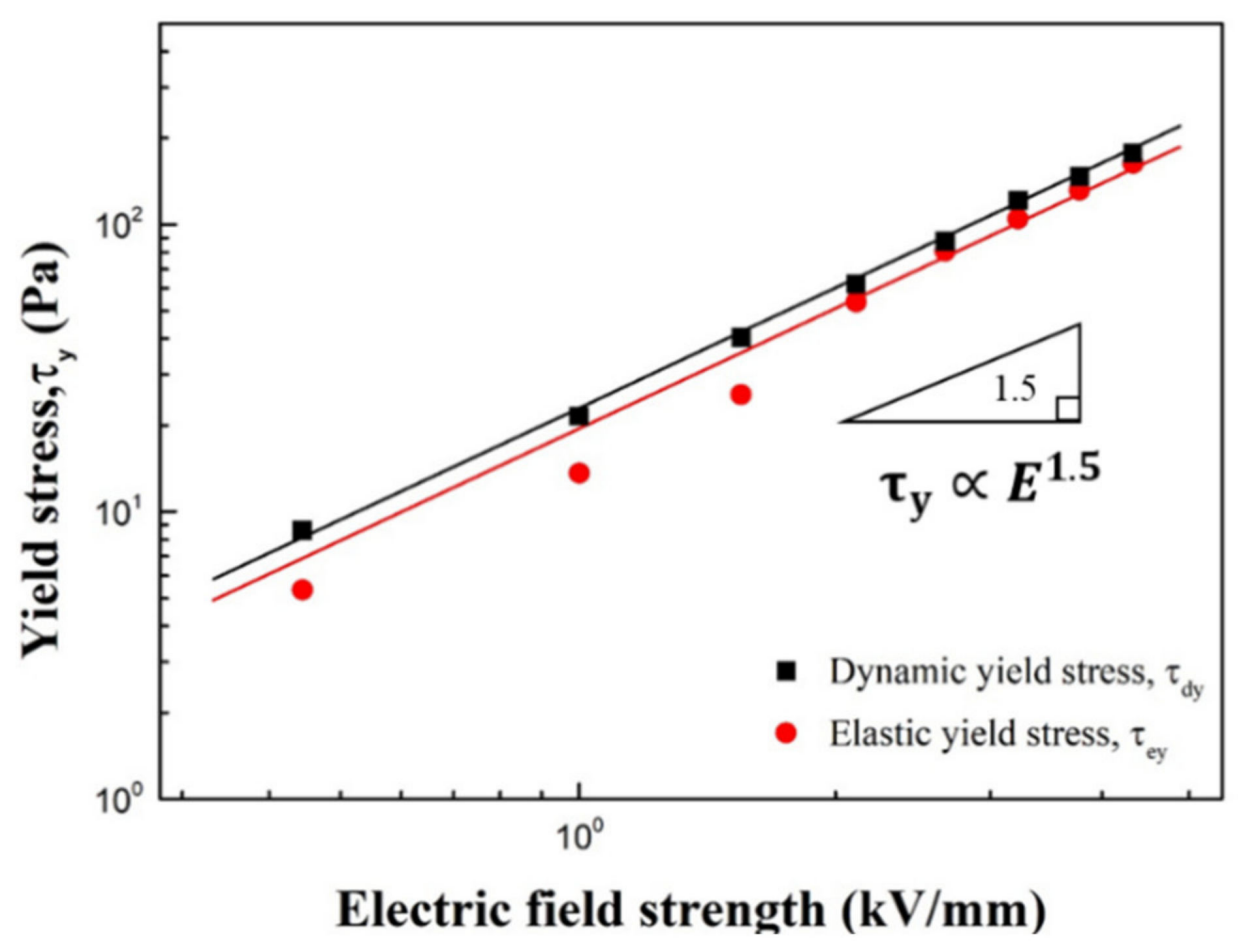
Elastic yield stress as a function of electric field strength of ZnFe_2_O_4_/PIn particles-based ER fluid.

Figure 9 shows the τ_dy_ deduced from the steady shear test and the elastic yield stress (τ_ey_) obtained from the dynamic test as a function of the electric field strength. τ_ey_ is the maximum stress that maintains elastic structure recovery when stress due to strain amplitude is removed. τ_ey_ can be defined as the maximum elastic stress or the value of the deflection point at which the slope change occurs [33,34].

Figure 9 was fitted using the power-law relation (Equation (2)), which implies a relationship with the E.

(2) $$\tau_{y}\propto {\;E}^{m}$$

where m is the slope of the log-log scale according to the electric field, E. Based on the dependence of the electric field strength for each ER material, m = 1.5 is proposed as the conductivity model and m = 2.0 is the polarization model. The slope (m) of the ZnFe_2_O_4_/PIn particle-based ER fluid was 1.5, corresponding to the conductivity model.

On the other, when this yield stress is compared with that of ZnFe_2_O_4_/polyaniline-based EMR fluid, it showed a slope of 1.5 corresponding to the conductivity model in the same way as the ZnFe_2_O_4_/PIn-based EMR fluid. On the other hand, the yield stress corresponding to each electric field is higher in ZnFe_2_O_4_/PIn, indicating that the ER effect is excellent in this study [35]. In addition, compared to the emulsion-polymerized PIn nanoparticle-based ER fluid of the maximum electric field strength of 2.5 kV/mm, the ZnFe_2_O_4_/PIn particle-based ER fluid in this study could tolerate 4.0 kV/mm because the thin layer of PIn in the core-shell structure possesses a better semi-conducting conductivity value for the ER fluid [36].

**Figure 10:**
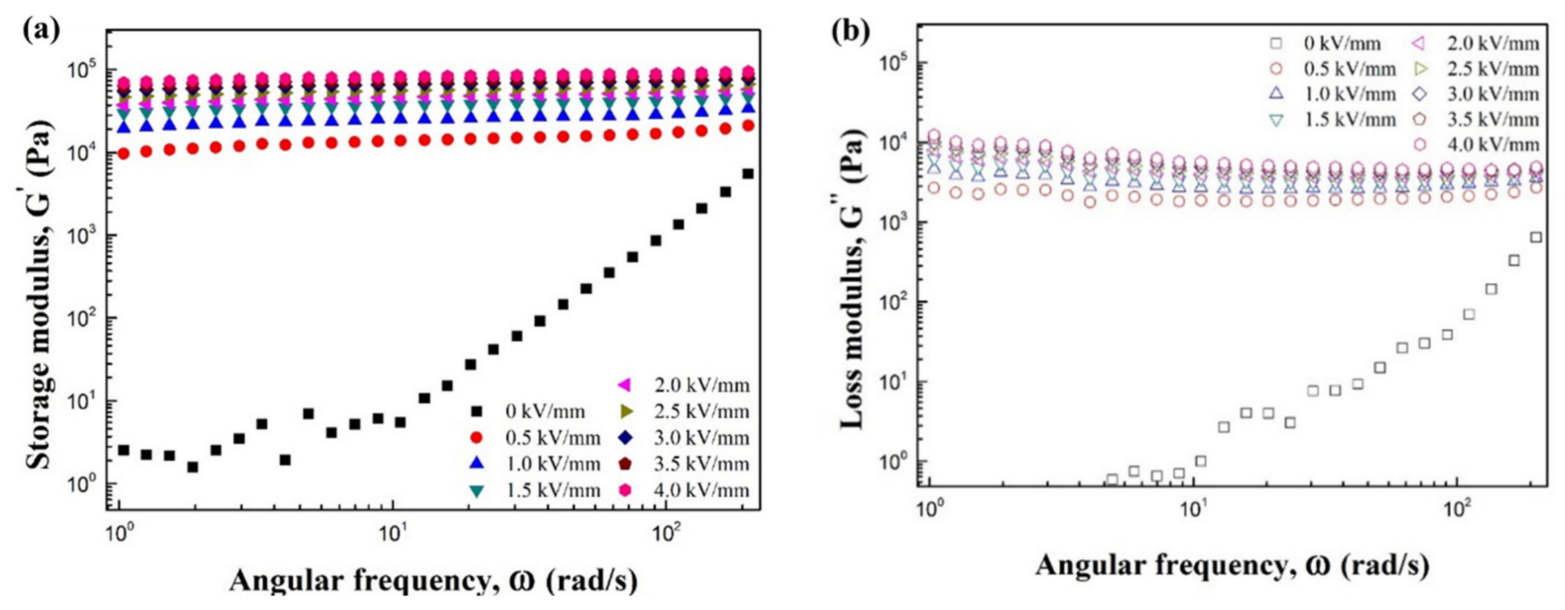
Storage modulus (**a**) and loss modulus (**b**) as a function of angular frequency of ZnFe_2_O_4_/PIn based ER fluid with electric field strength.

Figure 10 shows G′ and G″ measured under various electric fields as applied with the ω varying from 1 to 200 rad/s. The dynamic frequency test was performed by fixing the strain of 0.01% of the LVE region defined through the amplitude strain test as discussed previously. Without an applied *E*, both G′ and G″ values gradually increase as the angular frequency increases, showing a liquid-like behavior. Under an applied *E*, the G′ becomes larger than the G″ in the overall angular frequency range, resulting in a solid-like behavior, and all moduli have a constant value and are observed as parallel regions. In addition, as the electric field increases, the strength of the interaction between neighboring particles in the ZnFe_2_O_4_/Pin-based ER fluid increases, resulting in a more rigid chain structure and gives the fluid a higher storage modulus [37].

**Figure 11:**
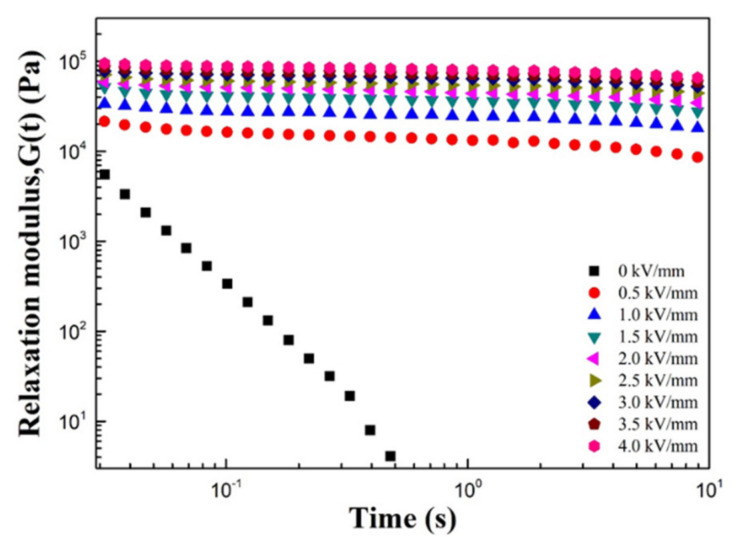
Relaxation modulus of ZnFe_2_O_4_/PIn particles-based ER fluid with varying time under electric field strength.

Figure 11 presents the relaxation modulus (G(t)) over time under an applied electric field. The relaxation modulus is calculated by substituting the value of the modulus at a certain frequency range, obtained through the angular frequency test, into the Schwarzl equation (Equation (3)) [38].

(3) $$G\left(t\right)\;≅\;G\prime \left(\omega \right)\;-\;0.566G\prime \prime \left(\omega /2\right)\;+\;0.203G\prime \prime \left(\omega \right)$$

The phase change from fluid-like to solid-like can be identified through the observed difference in the relaxation modulus of the ZnFe_2_O_4_/Pin-based ER fluid when the *E*s are applied. At the *E* of 0 kV/mm, $G\left(t\right)$ decreases rapidly over a short timeframe, indicating the liquid-like behavior of the ZnFe_2_O_4_/Pin-based ER fluid. In contrast, under the *E*, the stress relaxation of the ER fluid is not observed, and a plateau region is maintained at a stable value of G(t). This is the result of solid-like properties manifested by the strong chain formation of the ZnFe_2_O_4_/Pin-based ER fluid under the *E* [39,40].

**Figure 12:**
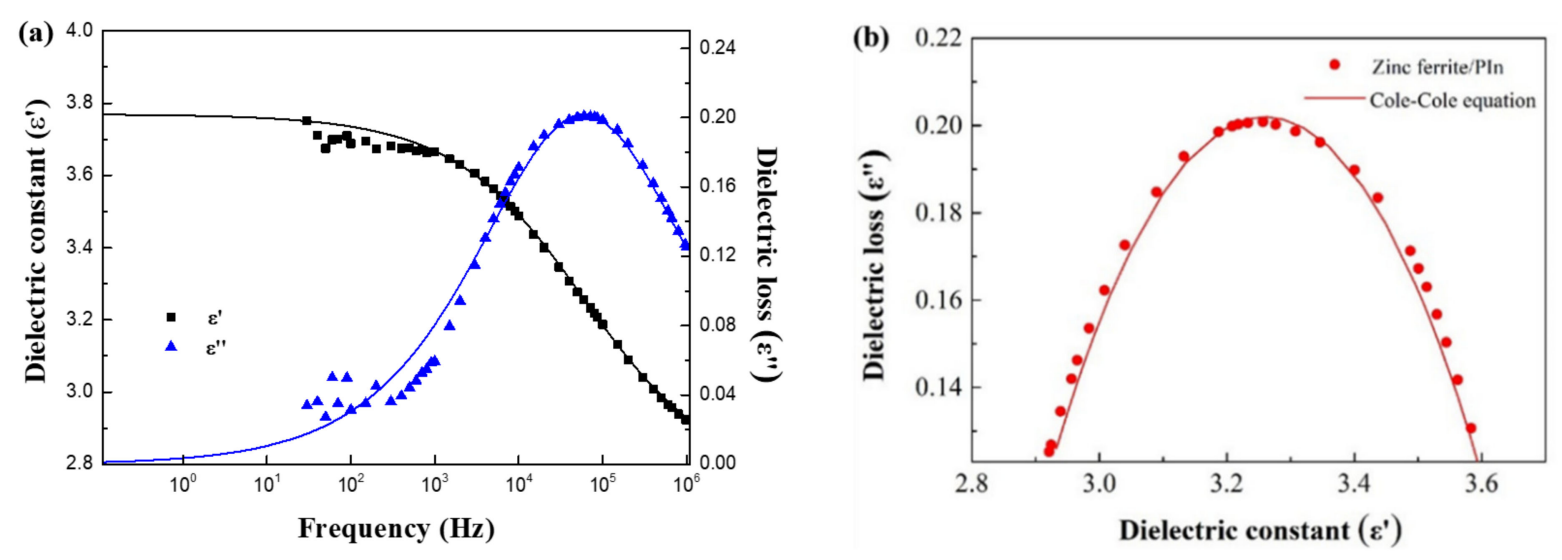
Dielectric spectra (**a**) and the Cole-Cole curve (**b**) of ZnFe_2_O_4_/PIn particles based ER fluid.

The following study was conducted to determine the genetic factors that influence the ER effect of ZnFe_2_O_4_/PIn particle-based ER fluid. The interfacial polarization of particles under an applied *E* is closely related to the electrostatic interaction that induces the chain structure inside the fluid; thus, the dielectric properties are investigated. Figure 12a presents the spectrum showing the ω dependence of the dielectric constant and dielectric loss in the angular frequency range of 20 Hz–1 MHz. As the angular frequency increases, ε′ gradually decreases, and ε″ exhibits a behavior with a peak at approximately 60,000 Hz. Figure 12b shows a Cole-Cole plot of the dielectric material. The solid lines shown in Figure 12a,b are all fitted by applying the Cole-Cole model (Equation (4)) and were consistent with the experimental dielectric data.

(4) $$\varepsilon^{*}=\;\varepsilon \prime +i\varepsilon \prime \prime =\varepsilon_{\infty }+\frac{\Delta \varepsilon }{1+{\left(i\omega \lambda \right)}^{1-\alpha }}$$

where ε* represents the complex dielectric constant as a function of ω, ε′ represents the dielectric constant, ε′ represents the dielectric loss, and ε_∞_ represents the permittivity at infinite frequencies. Δε is the difference between $\varepsilon_{0}$ and $\varepsilon_{\infty }\left(\varepsilon_{0}-\varepsilon_{\infty }\right)$, which is the limit dielectric constant, and is the dielectric relaxation strength. λ is the relaxation time ($1/2{\pi \omega }_{\max}$, $\omega_{\max}$ is the frequency at the peak where ε″ has the maximum value), and α (0 ≤ α < 1) is the Cole-Cole parameter [41,42]. Table 1 lists several parameters related to the Cole-Cole equation corresponding to the dielectric properties of the ZnFe_2_O_4_/PIn particle-based ER fluid.

## 4. Conclusions

In this study, ZnFe_2_O_4_, a soft magnetic particle with a spherical morphology, was synthesized using a simple solvothermal method. ZnFe_2_O_4_/PIn particles were subsequently prepared by coating the ZnFe_2_O_4_ particles with PIn via oxidation polymerization. Performing acid treatment on the surface of ZnFe_2_O_4_ acting as a core facilitated interaction with the polymer serving as a shell. To confirm the successful synthesis of ZnFe_2_O_4_/PIn particles, their morphologies and crystal structures were measured using SEM, TEM, and XRD. In addition, the chemical compositions of the particles were analyzed using FT-IR. The rheological characteristics of the ER fluids were tested using a rheometer. Dynamic oscillation tests were focused under an electric field, wherein the particle chains produced in response to electric fields exhibited excellent reversible state changes from liquid to solid. The modulus values increased with an increase in electric field, which also characterized the transition to solid behavior. Under an applied *E*, the elastic yield stress increased proportionally to a power of 1.5. The prepared fluid exhibited a response to electric fields and stable rheological properties of typical ER fluids. In addition, the dielectric characteristics of the ZnFe_2_O_4_/Pin-based ER fluids were tested using an LCR meter. This study demonstrates that ZnFe_2_O_4_/PIn ER fluids are easily synthesized and are useful as a smart fluid.

## Figures and Tables

**Table 1:** Fitting parameters of the Cole-Cole model Obtained from the ZnFe_2_O_4_/PIn particles based ER fluid.

Sample	Parameters				
	$\varepsilon_{0}$	$\varepsilon_{\infty }$	Δε	α	λ (ms)
ZnFe_2_O_4_/PIn	3.77	2.75	1.02	0.52	0.0027

## Data Availability

Data available on request.
